# Aptamer-coated track-etched membranes with a nanostructured silver layer for single virus detection in biological fluids

**DOI:** 10.3389/fbioe.2022.1076749

**Published:** 2023-01-10

**Authors:** Vladimir Kukushkin, Olga Kristavchuk, Evgeny Andreev, Nadezda Meshcheryakova, Olga Zaborova, Alexandra Gambaryan, Alexander Nechaev, Elena Zavyalova

**Affiliations:** ^1^ Osipyan Institute of Solid State Physics RAS, Chernogolovka, Russia; ^2^ Joint Institute for Nuclear Research, Dubna, Russia; ^3^ Lomonosov Moscow State University, Moscow, Russia; ^4^ Chumakov Federal Scientific Centre for Research and Development of Immune and Biological Products RAS, Moscow, Russia

**Keywords:** aptamer, aptasensor, influenza, membrane, SERS, virus

## Abstract

Aptasensors based on surface-enhanced Raman spectroscopy (SERS) are of high interest due to the superior specificity and low limit of detection. It is possible to produce stable and cheap SERS-active substrates and portable equipment meeting the requirements of point-of-care devices. Here we combine the membrane filtration and SERS-active substrate in the one pot. This approach allows efficient adsorption of the viruses from the solution onto aptamer-covered silver nanoparticles. Specific determination of the viruses was provided by the aptamer to influenza A virus labeled with the Raman-active label. The SERS-signal from the label was decreased with a descending concentration of the target virus. Even several virus particles in the sample provided an increase in SERS-spectra intensity, requiring only a few minutes for the interaction between the aptamer and the virus. The limit of detection of the aptasensor was as low as 10 viral particles per mL (VP/mL) of influenza A virus or 2 VP/mL per probe. This value overcomes the limit of detection of PCR techniques (∼10^3^ VP/mL). The proposed biosensor is very convenient for point-of-care applications.

## 1 Introduction

Surface-enhanced Raman spectroscopy (SERS) is a spectroscopic method, which implies multiple amplification of Raman scattering intensity by nanostructured interfaces of metals and dielectrics. Raman spectra are characteristic for a chemical structure of compounds; thus, they are used for identification purposes. It is possible to decode complex mixtures consisting of several different compounds; this feature allows simultaneous identification of several analytes in the one pot (Kukushkin et al., 2017; Tahir et al., 2021). Besides the Raman spectra, the fluorescence spectra are also enhanced (surface-enhanced fluorescence, SEF), but they contain broad peaks instead of a set of sharp peaks in Raman spectra (Belik et al., 2018; Sultangaziyev and Bukasov, 2020). This type of spectroscopy can be used to determine only one fluorescent compound in the presence of multiple non-fluorescent molecules, so that it is difficult to decode a mixture of fluorescent compounds.

Surface-enhanced Raman/fluorescent spectrometry is widely used as an analytical tool in biosensors providing ultra-low limits of detection in a biological media (Ambartsumyan et al., 2020; Eskandari et al., 2022; Kukushkin et al., 2019; Perumal et al., 2021; Sultangaziyev and Bukasov, 2020; Tahir et al., 2021; Ye et al., 2022; Zavyalova et al., 2022). Biomacromolecules *per se* are rather complex for specific determination as thousands of peptides and proteins are composed of the same set of amino acids. Raman spectroscopy of biomacromolecules does not allow sequence determination while revealing composition of some surface epitopes only. The specific determination can be provided using recognizing molecules such as antibodies or nucleic acid aptamers labeled with Raman-active labels, tags or fluorophores (Ambartsumyan et al., 2020; Lee et al., 2015; Lin et al., 2014; Zavyalova et al., 2022). Nucleic acid aptamers are useful for application in SERS as they are small (2–5 nm) chemically synthesized macromolecules with a capability of site-specific modification such as thiol groups, Raman-active labels, fluorophores etc. (Adachi and Nakamura, 2019; Ni et al., 2021). Moreover, it is possible to introduce several modifications simultaneously. It is especially significant for analyte determination with low limit of detection (Kraemer et al., 2011). An example of usage of double modified aptamer in SERS-based technique is provided in our recent work (Kukushkin et al., 2022a). In that work the aptamer was immobilized onto the metal surface through a thiol group, whereas the Cyanine-3 dye was used as a reporter which signal decreased when the coronavirus (SARS-CoV-2 virus) bound to the aptamer.

Biological media are challenging for SERS-based sensors as non-specific adsorption of biomolecules drastically decreases the signals from the target molecules. For example, blood plasma is to be diluted at least 1,000-times for colloidal SERS-sensors (Zhdanov et al., 2022a), whereas the effect from impurities of the allantoic fluid was observed even after 10^6^-times dilution with a buffer (Zhdanov et al., 2022b). Membrane filtration makes the task much easier for decreasing the concentration of non-specific compounds. For example, influenza A virus was detected with 10^5^-times lower limit of detection due to the membrane filtration (Zhdanov et al., 2022b). Porous filters that provide SERS and/or SEF effects are high interest (Kristavchuk et al., 2017; Feng et al., 2020). The filters can be used for both prefiltration of the sample (Laserna et al., 1988; Muniz-Miranda et al., 1997; Zhdanov et al., 2022b); and as flow-through chip with simultaneous filtration and signal enhancement capabilities (Taurozzi and Tarabara, 2007; Feng et al., 2020; Serebrennikova et al., 2022).

Moderate robustness of the aptamer-coated track-etched membranes with nanostructured silver layer for influenza A/B virus determination was shown in our recent work (Kukushkin et al., 2022b). SERS-active layer made up of silver nanoislands only appeared to be unstable in biological fluids, especially after the functionalization with the aptamer and the further virus treatment. Aptamer-functionalized nanoparticles were flushed out from the surface by the influenza A virus as the interaction between aptamer and virus was stronger than the adhesion of nanoparticles to the membrane. Simultaneous deposition of chromium and silver on the track-etched membrane produced stable coating that enhanced fluorescence signal from Cyanine-3-labeled aptamer but provided no SERS. The lowest determined concentration of influenza A virus was 4·10^–4^ HAU/mL.

Here we used the same track-etched membrane with chromium and silver coating with a modified aptasensor design. However, the working principle of the sensor is completely different. We used double-labeled aptamer for influenza A virus with thiol and Cyanine-3 dye, instead of the previous arrangement with two aptamers, thiol-modified aptamer and Cyanine-3-labeled aptamer. The decrease of the participants in the final complex can decrease the limit of detection. Another important process is approaching of the SERS-active compound to the surface providing the SERS spectra. Analyte-dependent changes of surface-enhanced Raman or fluorescence spectra are tested in this work.

## 2 Materials and methods

Inorganic salts were purchased from AppliChem GmbH (Darmstadt, Germany) and Sigma-Aldrich (St. Louis, MO, United States). The following modification of DNA aptamer RHA0385 was synthesized by Synthol (Moscow, Russia): (SH-dT)-5′-TTGGGTTATTTTGGGAGGGC-GGGGGTT-3′-Су3. The solutions were prepared in ultrapure water with resistivity of 18.2 MΩ.

### 2.1 Viruses

Influenza A virus from H7N1 subtype (A/chicken/Rostock/45/1934) and influenza B virus (B/Victoria/2/1987) were propagated in the allantoic cavity of 10-day-old embryonated specific pathogen-free chicken eggs. Eggs were incubated at 37°C, cooled at 4°C for 48 h post-infection, and harvested 16 h later. The study design was approved by the Ethics Committee of the Chumakov Institute of Poliomyelitis and Viral Encephalitides, Moscow, Russia (Approval #4 from 2 December 2014). Viruses were inactivated *via* the addition of 0.05% (v/v) glutaric aldehyde and preserved *via* the addition of 0.03% (w/v) NaN_3_, and stored at +4°C. The viral-dependent agglutination of red blood cells was not impaired by the inactivation procedure; thus, the inactivation procedure did not disrupt the conformation of the hemagglutinin protein, a target of DNA aptamer RHA0385. Previously, we have shown that aptamer RHA0385 inhibits viral-dependent agglutination of red blood cells due to binding of influenza A hemagglutinin as a part of the inactivated influenza A viruses (Bizyaeva et al., 2021). These data confirm the possibility of usage of inactivated viruses in further investigation.

Viral titer was determined using a hemagglutination assay. A measure of 50 µl of solutions of the viruses diluted two times step-by-step in a phosphate-buffered saline was placed in a V-shaped 96-well microtiter plate in a volume of 50 µl. Then, 50 µl of 0.5% chicken red blood cells in the phosphate buffered saline was added to the well. The plate was kept in the refrigerator at 4°C for 1 h. Then, the hemagglutination titer was estimated as the maximal dilution of the virus that did not cause the precipitate of red blood cells; this well contained 1 HAU of the virus in the probe.

### 2.2 Determination of viral particles in the sample

Nanoparticle Tracking Analysis (NTA) was conducted using a ZetaView^®^ PMX420-QUATT instrument (Particle Metrix GmbH, Germany), while the data were analyzed by ZetaView NTA software. Operating instructions of the manufacturer were followed before calibrating the instrument with a known concentration of 100 nm polystyrene nanoparticles (Applied Microspheres B.V., Netherlands). The standards were suspended in particle-free water, whereas the investigated samples were diluted 1:100 with phosphate buffered saline (PBS). Particles were counted and size-distributed at 10 cycles of 11 frames per cycle under sensitivity of 65 and a shutter value of 100.

### 2.3 SERS-membrane

Polyethylene terephthalate track-etched membrane with average pore diameter of 360 nm and pore density of 2.6·10^8^ cm^−2^ were produced according to previous work (Apel, 1995). The nanostructured surface was obtained by evaporating the metals with thin film deposition system NANO 38 (Kurt J. Lesker Company, Jefferson Hills, PA, United States). The layer of chromium was 10 Å, and the layer of silver was 80 Å. Both layers were evaporated under 8∙10^–7^ Torr with a deposition rate of 0.4 Å/s. Then the membrane was heated at 120°C for 6 min using a heating plate HP-20D-Set (Daihan Scientific, Singapore, Singapore).

Raman XY-topography of the surface of track membranes was performed with a step of 200 microns on an area of 2 × 2 mm. Thus, 100 spectra of the test substance (4-aminobenzenethiol) were recorded on each of the five measured membranes and five commercial substrates EnSpectr SERS. As a result of comparing the averaged intensities of the 1,142 cm^−1^ line from the test substance on the membrane and on the substrate, an absolute Q-factor value was calculated.

### 2.4 SERS aptasensor

The aptamer was folded into the active conformation by diluting in PBS buffer in concentration of 2 µМ with subsequent heating at 95°C for 5 min and cooling at room temperature. The aptamer solution was diluted in PBS buffer to concentration of 200 nM. The membrane was incubated in 200 nM aptamer solution for 15 min. A round fragment of the membrane with a diameter of 3 mm was placed onto the filter block of Corning Costar Spin-X centrifuge tube with cellulose acetate membrane filters, pore size 0.45 µm (Corning, NY, United States). Virus-containing allantois fluids were diluted 20–10^9^ -times in PBS buffer. 200 µL of the sample was filtered through the membrane using Microspin FV-2400 (Biosan, Riga, Latvia) for 2 min. SERS spectra were recorded for 400 ms with averaging 20 repeats using Raman spectrometer EnSpectr SERS R532 (Enhanced Spectrometry, Meridian, ID, United States) with laser wavelength of 532 nm. The diameter of the beam was 2 mm. Each experiment was repeated 3 times; the normalized SERS or SEF intensities were averaged. The limit of detection was calculated from the linear regression of SERS (SEF) intensities plotted *versus* logarithm of influenza concentration (the ascending part of the curve was used). The limit of detection was estimated as the concentration of the virus which increased the SERS (SEF) intensity by three standard deviations over the intensity in the blank sample. The limit of quantification was estimated as the concentration of the virus which increased the SERS (SEF) intensity by ten standard deviations over the intensity in the blank sample.

### 2.5 Scanning electron microscopy

Scanning electron microscopy (SEM) of the membrane samples was performed using electron microscope SU 8020 equipped with cold field cathode (Hitachi, Tokyo, Japan). The resolution and contrast of the images were improved by evaporating a layer of gold-palladium alloy with a thickness of 5 nm.

## 3 Results

### 3.1 Topology and Q-factor of SERS-active membrane

**FIGURE 1:**
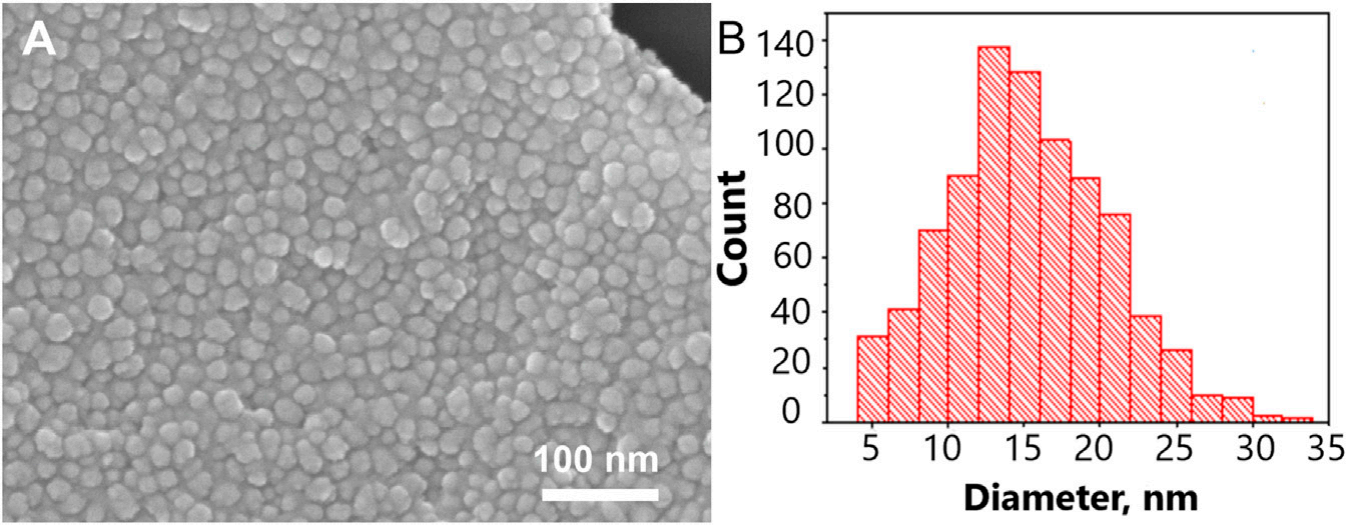
The nanostructured surface of the tracked-etched membrane. **(A)** Scanning electron microscopy of the surface. **(B)** The distribution diagram of a nanoparticle size on the track-etched membrane.

The size of metal nanoparticles onto the track-etched membrane was 14 ± 5 nm. The gaps between nanoparticles are nearly 2 nm. The surface is almost completely covered with nanoparticles. The distribution diagram is shown in Figure 1. To calculate the Q-factor of SERS-active membrane, a test substance 4-aminobenzeniol at a concentration of 10^–5^ g/mL was applied to a membrane with a silver nanostructure layer. This substance contains an SH-group and therefore remains on the surface of the membrane and does not leak through the pores, thereby making it possible to compare signals from the membrane and from a commercial substrate EnSpectr SERS (Enhanced Spectrometry, Inc., United States) with a known Q-factor, namely, Blue & Green Substrate (Enhanced Spectrometry, Inc., Meridian, ID, United States) with the enhancement factor of 2.2·10^6^. Comparing SERS signals from the test molecules under the same conditions of excitation and signal registration, it is possible to calculate the coefficient of membrane amplification. The Q-factor calculated in this way was 7·10^5^ (Supplementary Figure S1).

### 3.2 Functionalization of the membrane with the aptamer

RHA0385 aptamer was chosen as a recognizing element of the sensor, since it was shown to have high affinity and selectivity towards the influenza A virus (Zhdanov et al., 2022b). The dissociation constant of the complex between RHA0385 and influenza A hemagglutinin is 0.6 nM (Bizyaeva et al., 2021); and the dissociation constant of the complex between RHA0385 and influenza A virus is as low as 1.1 pM (Zhdanov et al., 2022b). The ultrahigh affinity to the viral particles is provided due to the multiple interactions between hemagglutinins and surface-anchored aptamers (so called ‘avidity’ that is the accumulated strength of multiple affinities of individual binding interactions).

Nanostructured silver layer of the membrane was functionalized with RHA0385 aptamer for influenza A virus with two modifications, namely thiol at the 5′-end and Cyanine-3 dye at the 3′-end. Thiol forms a covalent bond with silver nanoparticles providing stable aptamer coating. Cyanine-3 is a fluorophore and Raman-label which allows estimation of efficiency and stability of the aptamer coating. Cyanine-3 is referred further as a reporter. The conditions of membrane functionalization with the aptamer were optimized.

The change of aptamer concentration from 20 nM to 2 µM increased the surface-enhanced fluorescence (SEF) and surface-enhanced Raman spectroscopy (SERS) of the reporter monotonously (Supplementary Figure S2). Similarly, time of incubation affects intensity magnitude of SEF and SERS spectra; the maximal intensities were achieved for 15–45 min of incubation in 200 nM solution of the aptamer. One more variable is a buffer for the aptamer solution. Water, PBS buffer and tris-nitrate buffer (10 mM tris-HCl, 140 mM NaNO_3_ and 10 mM KNO_3_) were tested. The highest SEF intensity was achieved for the membrane functionalization with the aptamer solution in PBS.

**FIGURE 2:**
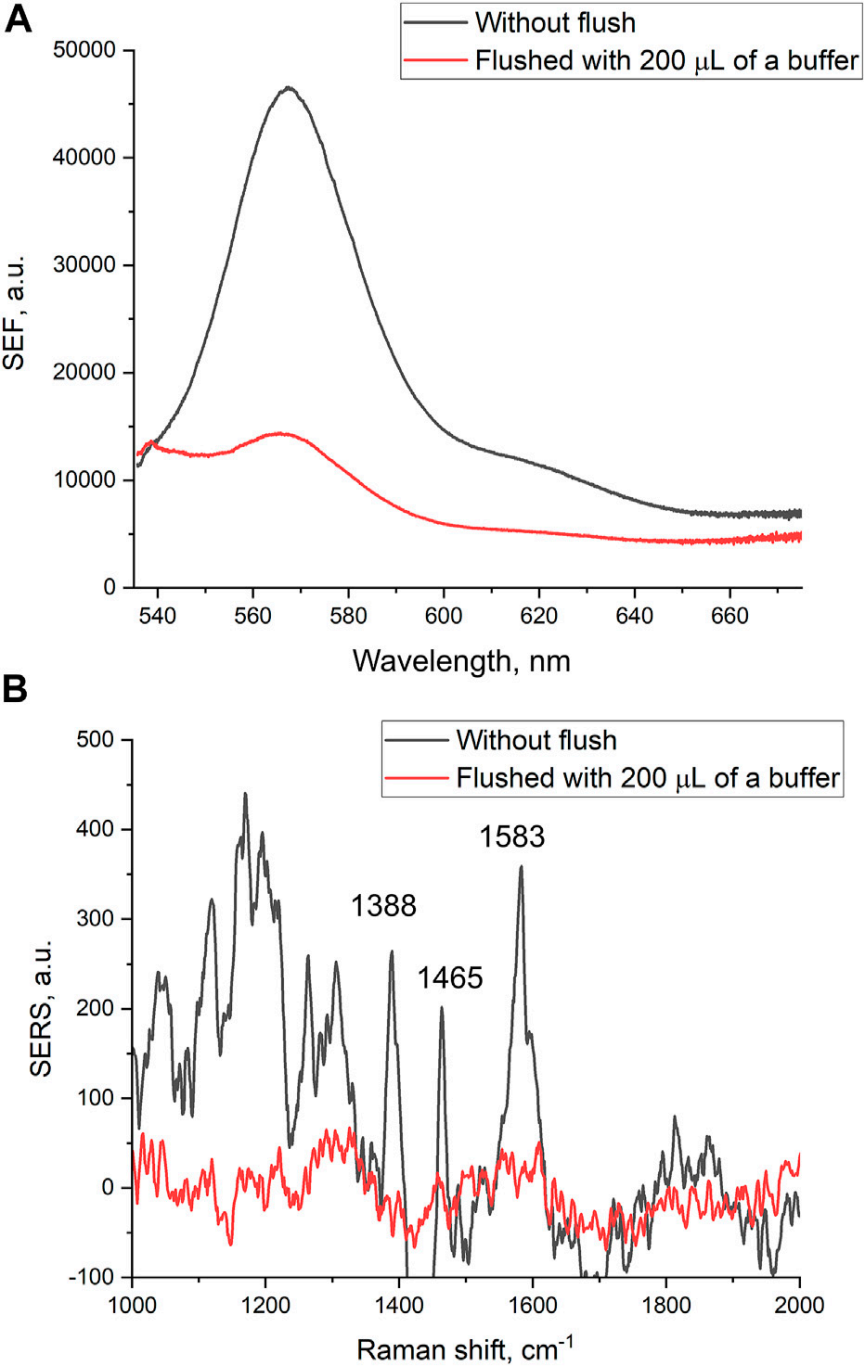
Surface-enhanced fluorescence **(A)** and surface-enhanced Raman **(B)** spectra of Cyanine-3 reporter obtained from the track-etched membrane functionalized with the aptamer before and after the filtration of phosphate-buffered saline.

**FIGURE 3:**
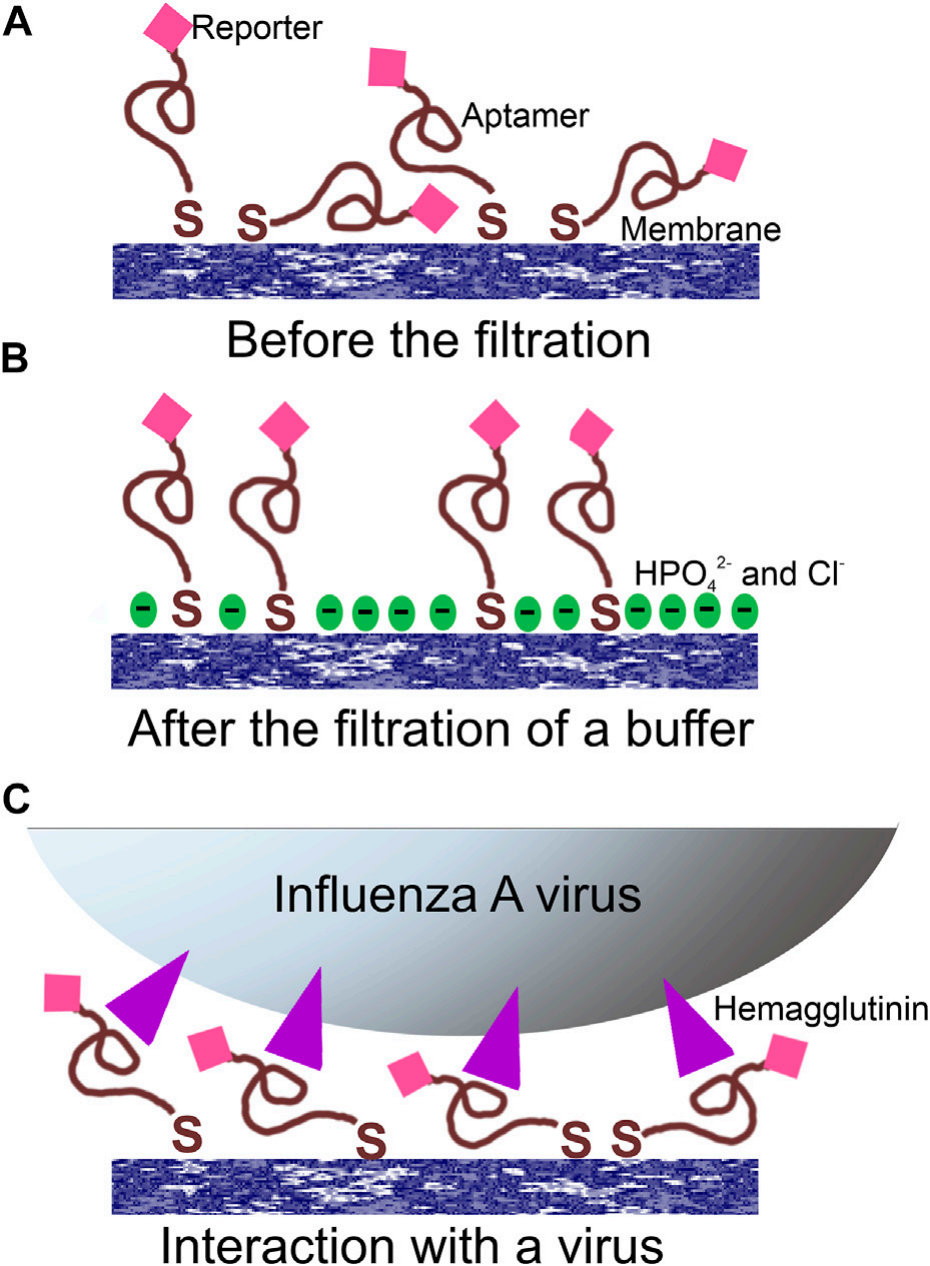
Proposed operating principle of the aptasensor. **(A)** Random orientation of the aptamer with the reporter toward the surface; **(B)** PBS filtration modified the surface providing a negatively charged surface with increased distance between the reporter and the surface; **(C)** the filtration of the virus provided specific interactions between aptamers and viral particles orienting the reporter in the common manner near the surface.

Filtration through the membrane also affects intensities of SEF and SERS spectra of the reporter. The intensities of both SEF and SERS spectra were decreased substantially after the filtering of 200 µl of PBS (Figure 2). The decrease in the intensities of the spectra was not associated with the destruction of nanostructured metal layer. Scanning electron microscopy revealed that the metal layer retained after the filtration (Supplementary Figure S3 and Figure 3A). Thу stability of the coating is maintained due to chromium layer, as in our previous work in the absence of chromium layer, the silver nanoparticles were flushed out from the membrane providing no SERS or SEF spectra of the reporter (Kukushkin et al., 2022b).

Additionally, the performance of the sensor was estimated for 20 nM, 200 nM and 2 µM concentration of the aptamer. The treatment with influenza A virus changed the SERS and SEF intensities non-monotonously. The membrane treated with the aptamer concentration of 200 nM provided the increase in SERS and SEF in the presence of the virus. Whereas the membrane treated with the aptamer concentration of 2 µM provided the decrease in the spectrum intensity (Supplementary Figure S2). Incubation for 15 min and concentration of the aptamer of 200 nM in PBS were chosen as the optimal conditions for influenza A detection.

A possible explanation of the SERS and SEF signal dependencies is connected with the orientation of the reporter relative to the surface (Figure 3). A modification of the surface of silver nanoparticles during the filtration of the buffer could cause the decrease SERS and SEF intensities due to desorption of Cy3-label from the surface (Figure 3B). The modification of the surface might be attributed to the oxidation of the silver and further adsorption of chlorides and phosphates from PBS buffer. On contrary, the hemagglutinin of influenza A virus binds G-quadruplex core of the aptamer (Bizyaeva et al., 2021) positioning the reporter near the surface (Figure 3C). Surface-enhanced spectra depend strongly on the distance between the reporter and the surface (Kumari et al., 2015). Thus, analyte-dependent change of the reporter positioning can be used to create a sensor.

The additional experiment was performed to ensure that the concept can be translated to other viruses. The concentration dependence of SERS intensity of TAMRA label conjugated with thiolated aptamer to respiratory syncytial virus (RSV) is nearly the same for the samples with filtration of PBS buffer and respiratory syncytial virus strain A2 (Supplementary Figure S4). Namely, the membrane treated with the aptamer concentration of 200 nM provided the increase in SERS and SEF in the presence of RSV. Whereas the membrane treated with the aptamer concentration of 2 µM provided the decrease in the spectrum intensity. The complete change of the virus type, aptamer sequence and the reporter did not change the operating principle of the sensor.

### 3.3 Membrane-based aptasensor for influenza a virus

Aptamers provide high affinity and selectivity. However, immobilization and modification can affect both affinity and specificity. This effect was shown for different aptamers, including aptamers to S-protein of SARS-CoV-2, thrombin, epidermal factor growth receptor (Zavyalova et al., 2020; Zhdanov et al., 2021; Grabovenko et al., 2022). The structure-affinity relationship for the aptamer RHA0385 was studied thoroughly. G-quadruplex core is a main element that provides high affinity of RHA0385 to hemagglutinin of influenza A virus. 5′- and 3′-ends of the aptamer can be truncated or modified retaining the affinity of the aptamer (Novoseltseva et al., 2020; Bizyaeva et al., 2021). The key problem is to provide analyte-dependent changes of surface-enhanced Raman or fluorescence spectra of the reporter. In the previous aptasensor architectures, the signal increased when the ternary complex thiol-modified aptamer-virus-labelled aptamer is assembled (Kukushkin et al., 2019; Kukushkin et al., 2022b); or decreased due to the removal of the aptamer from the SERS-active surface by the virus (Chen et al., 2020; Chen et al., 2022).

**FIGURE 4:**
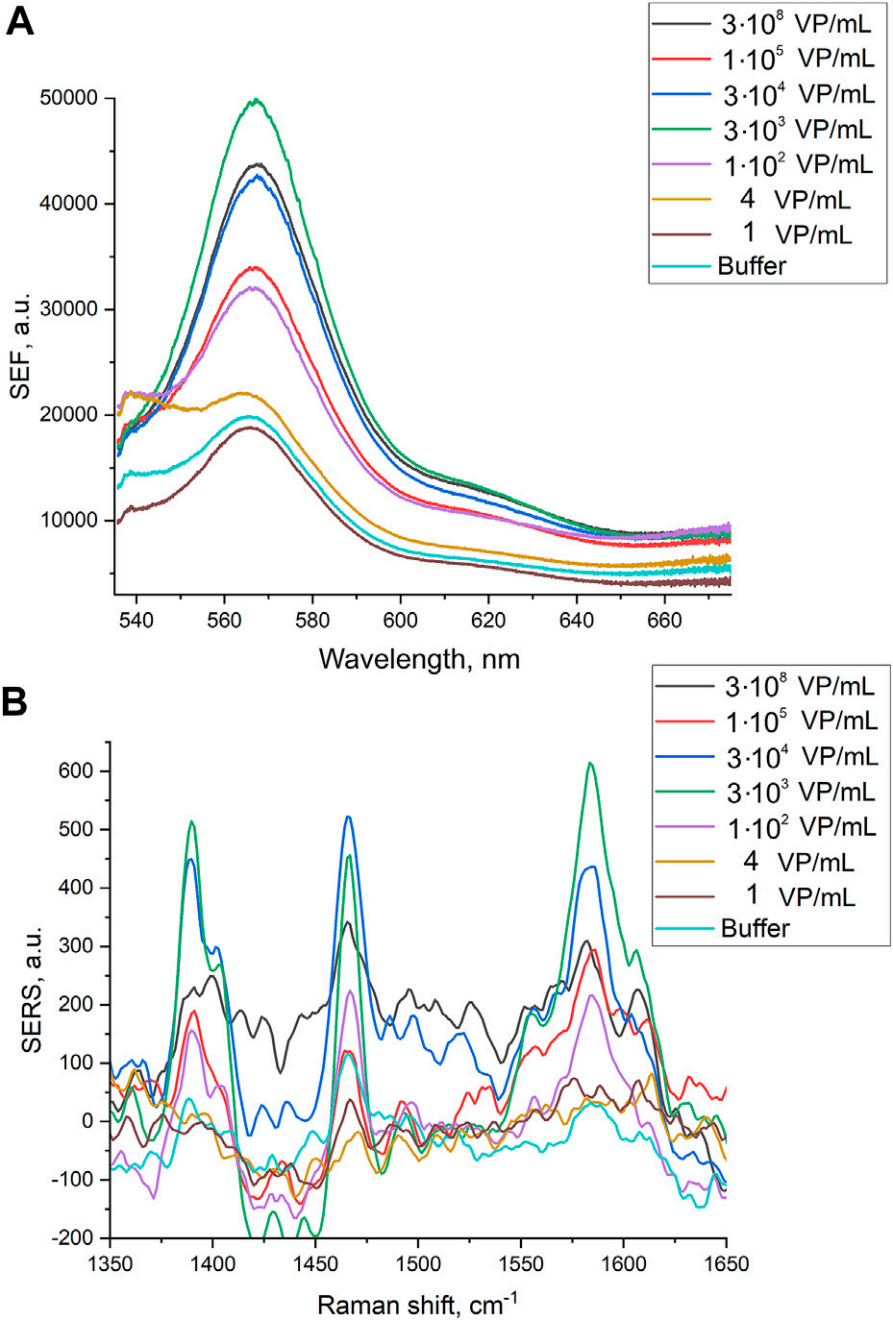
Surface-enhanced fluorescence **(A)** and surface-enhanced Raman **(B)** spectra of Cyanine-3 reporter obtained from the track-etched membranes functionalized with the aptamer after the filtration of the probes with different concentrations of influenza A virus.

**FIGURE 5:**
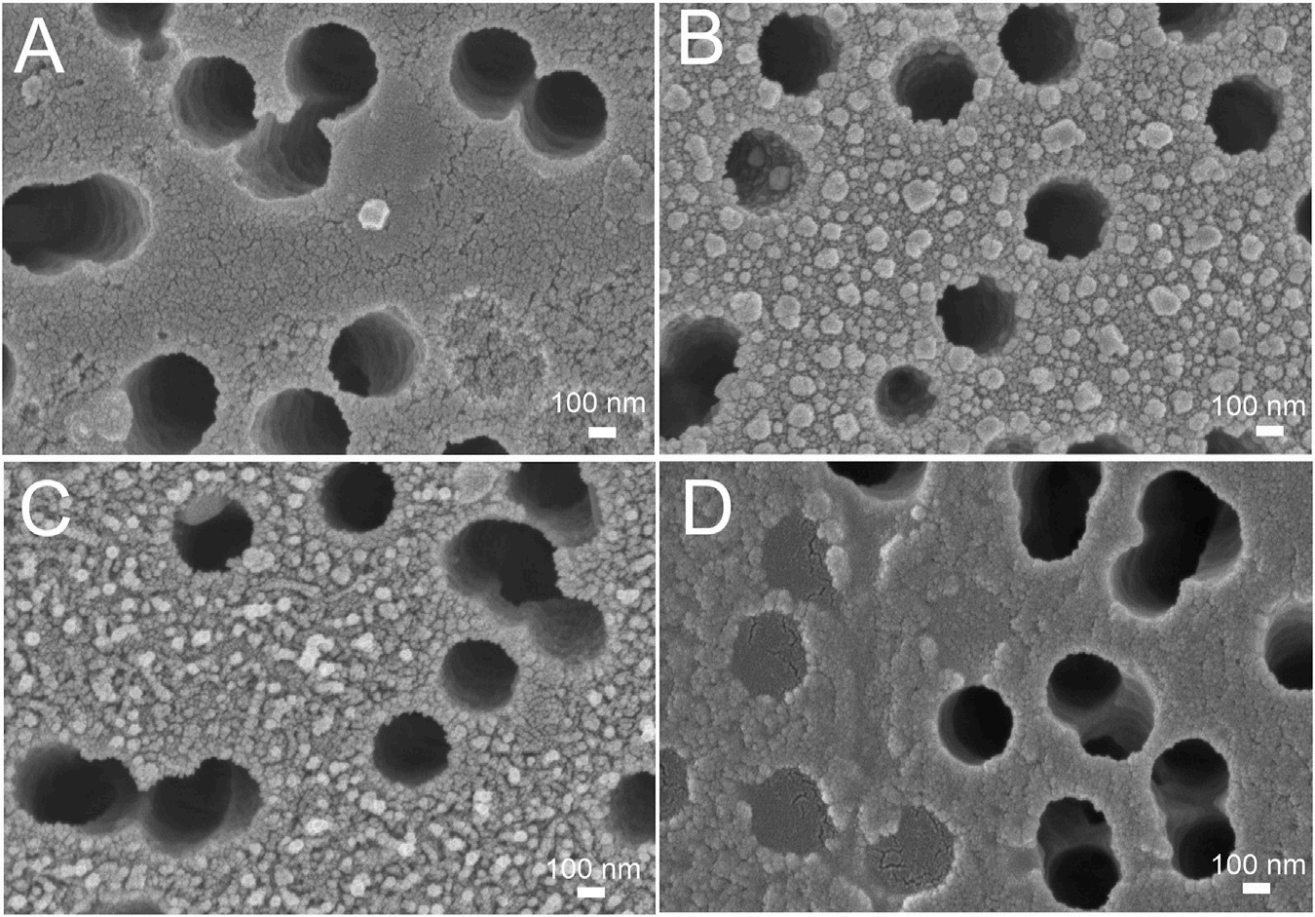
Scanning electron microscopy of the track-etched membranes functionalized with the aptamer after the filtration of phosphate-buffered saline **(A)**, 3·10^8^ VP/mL of influenza A virus **(B)**, 2·10^6^ VP/mL of influenza A virus **(C)**, and 30 VP/mL of influenza A virus **(D)**.

Here we tested an alternative setup where the 3′-end-labeled aptamer is adsorbed on the SERS-active surface due to thiol-modification at the 5′-end of the aptamer. The adsorbed aptamer provided SERS and SEF signals of Cyanine-3. The design of the aptamer (i.e. sites for incorporation of thiol and Cyanine-3) provided the increase in surface-enhanced Raman and fluorescence spectra intensities in the presence of the analyte (Figure 4) contrary to the empty samples. Scanning electron microscopy revealed changes in a surface topology in the presence of influenza A virus, especially at high viral titers (Figure 5). The surface became non-uniform with spherical nanoparticles with a diameter of 30–100 nm. Presumably, these objects were formed due to the interaction between nanoparticles and influenza A virus. Similar objects were observed onto the nanoisland silver substrates with clear distortion of the surface (Supplementary Figure S5). As it is shown further, this circumstance hinders the quantification of influenza A virus at a high viral titer.

### 3.4 Quantification of viral particles of influenza a virus

**FIGURE 6:**
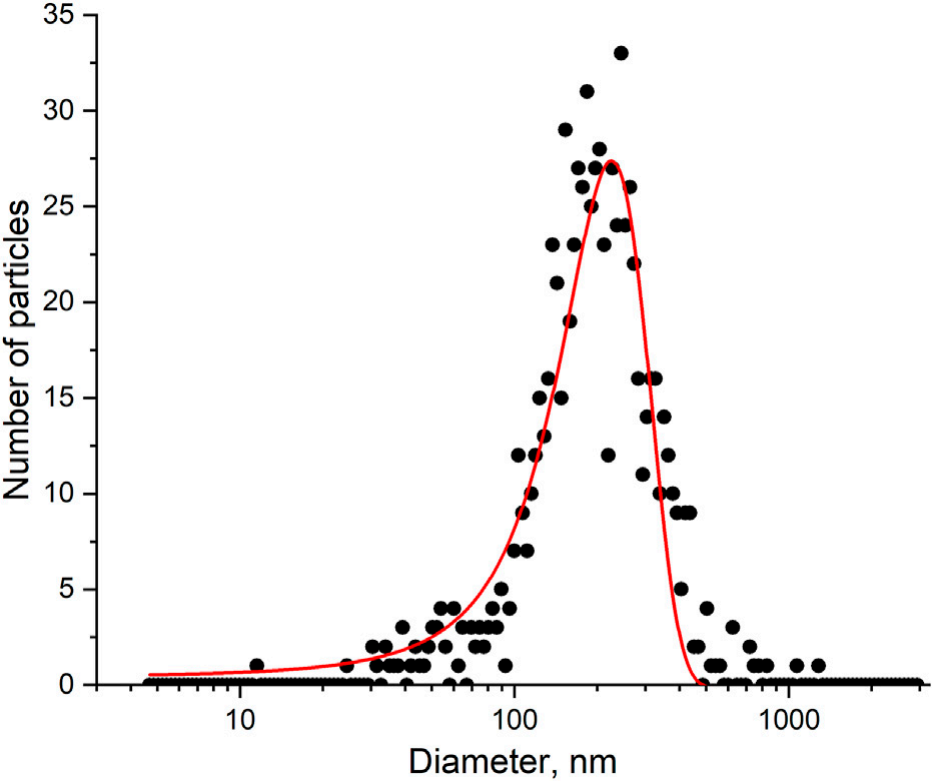
A nanoparticle tracking analysis of the influenza A virus sample. The experimental size distribution of the nanoparticles and its approximation.

There are many ways to estimate the quantity of influenza A virus. The most popular units are copies of viral genome determined by PCR, hemagglutination units (HAU) determined by the interaction with red blood cells, and plaque forming units (pfu) determined by a cell viability assay. These values can be recalculated into viral particles using several published correlations; however, this procedure provides a rough estimation. Here we performed a nanoparticle tracking analysis to estimate the quantity of nanoparticles in the sample. This technique physically calculates nanoparticles in the sample with a simultaneous estimation of their size. The distribution is shown in Figure 6. The virus concentration was estimated as 6 10^9^ VP/mL with a mean diameter of 160 ± 30 nm.

### 3.5 Performance of the membrane-based aptasensor for influenza a virus

**FIGURE 7:**
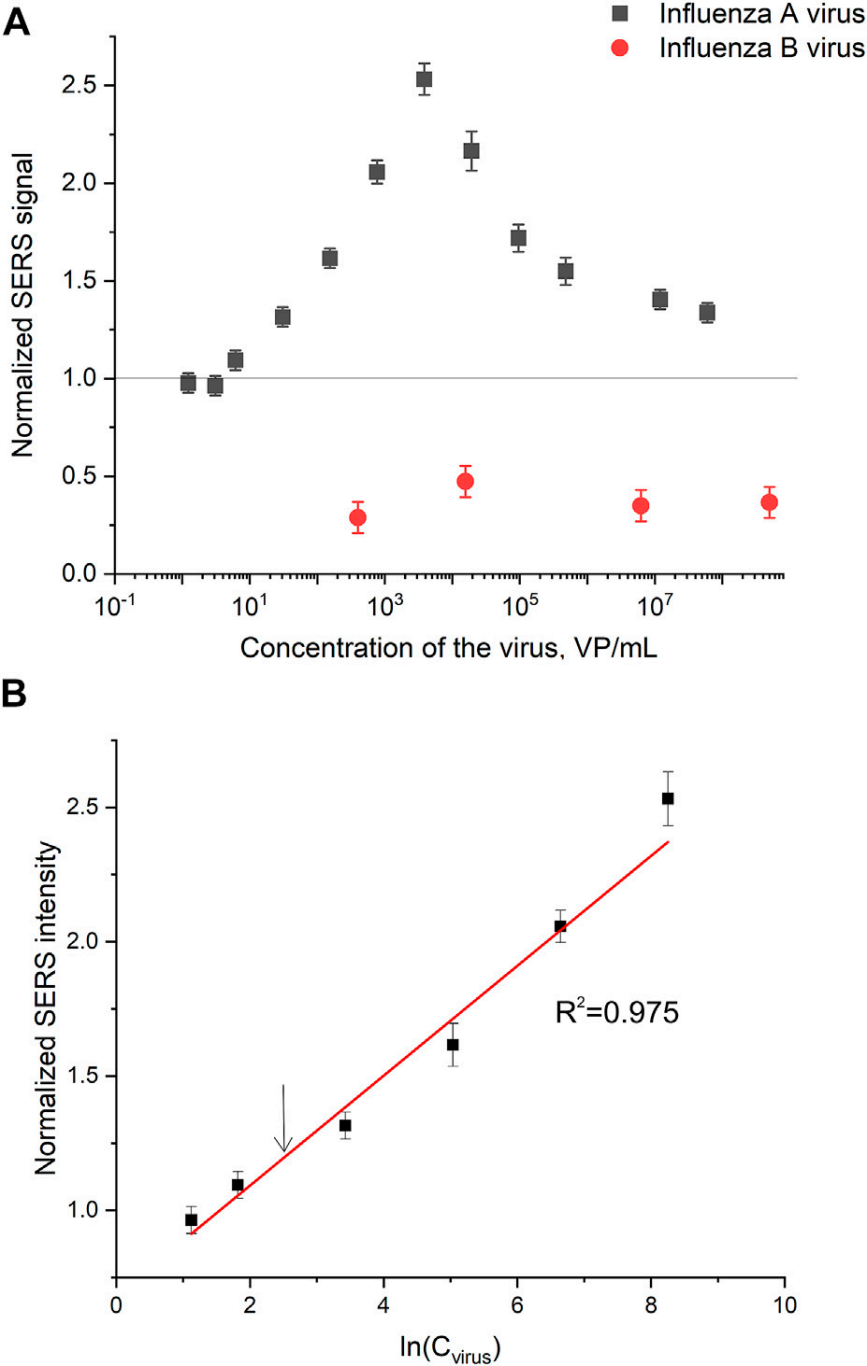
A concentration dependence of a normalized surface-enhanced Raman spectrum intensity of signal from Cyanine-3 reporter on the concentration of influenza A virus and influenza B virus **(A)**. The part of the dependence with a monotonous increase of the signal was used to calculate the limit of detection that is indicated by an arrow **(B)**.

**FIGURE 8:**
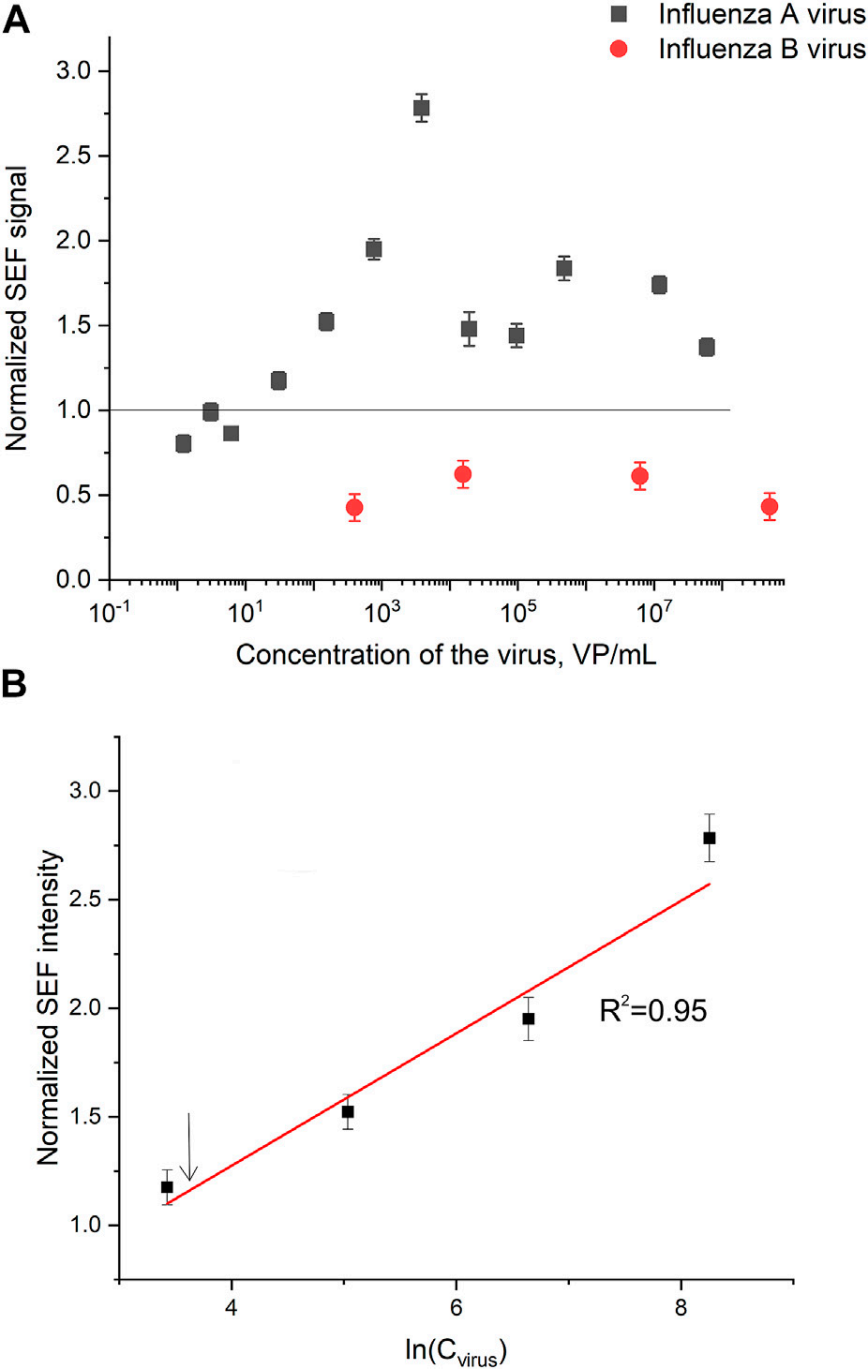
A concentration dependence of a normalized surface-enhanced fluorescence spectrum intensity of signal from Cyanine-3 reporter on the concentration of influenza A virus and influenza B virus **(A)**. The part of the dependence with monotonous increase of the signal was used to calculate the limit of detection that is indicated by an arrow **(B)**.

Normalized SERS and SEF intensities were used to estimate performance of the membrane-based aptasensor for influenza A virus. The intensities of the samples with influenza A virus were divided by the intensities in the control experiments without the virus. The closest counterpart of influenza A virus, influenza B virus, was used to estimate specificity of the sensor. Normalized SERS and SEF intensities were >1 for a wide concentration range of influenza A virus (Figure 7A; Figure 8A). Whereas the samples with influenza B virus had normalized SERS and SEF intensities ≤1. The normalized intensities of both SERS and SEF spectra increased monotonously from 1.0 to 2.8–3.0 in the range of influenza A concentrations of 0–3.8·10^3^ VP/mL (Figure 7B; Figure 8B). Further increase of influenza A virus led to the decrease of SERS and SEF normalized intensities down to 1.3–1.5. These sample are still ‘positive’ but the quantification of the concentrated samples is complicated. Scanning electron microscopy revealed significant distortions of the nanostructured surface of the aptasensors with a high content of influenza A virus (Figure 5). Obviously, the changes in the surface topology disrupted the monotonous dependence of the aptasensor. The limit of detection of the sensor was calculated as three standard deviations of SERS intensity at 1,587 cm^−1^ over the control without influenza A virus. The limit of detection for the SERS sensor was as little as 10 VP/mL (2 VP per probe) of influenza A virus; whereas the limit of detection for the SEF sensor 25 VP/mL of influenza A virus (5 VP per probe). These results are the best among published works (see the Discussion part for the detailed comparison). The limit of quantification was estimated as 33 VP/mL and 80 VP/mL for SERS and SEF dependencies, respectively. The quantification is rather complicated as the dependence is non-linear.

## 4 Discussion

The development of SERS substrates is ongoing aiming to combine high enhancement factors with simplicity of fabrication of the substrate and its application. A huge variety of solid substrates obtained with a lithography (Bilgin et al., 2022), multilayer deposition (Chen et al., 2020; Yeh et al., 2020; Paria et al., 2022; Ye et al., 2022) or etching (Zhang et al., 2019) were applied for virus identification. This type of substrates can provide a high enhancement factor, a high degree of homogeneity of the surface, but they are rather complex in manufacturing requiring multiple procedures. An alternative approach is to decorate the surface with SERS-active nanoparticles. Viruses were detected using porous carbon films (Luo et al., 2017) or even cellulose paper (Santos et al., 2022) decorated with nanoparticles. This approach provides simplification of SERS substrate fabrication, price reduction and the easy handling of the substrate. The substrates can be sliced into any shape; and they are flexible adopting any geometry of the sensor.

Here we used membranes decorated with silver nanoparticles. The polyethylene terephthalate track-etched membranes can be obtained with a pore diameter from 10 nm to 1 µ providing a wide range of applications. In particular, we performed concentrating of the influenza A viruses and their purification from any molecules and macromolecules (Zhdanov et al., 2022b). SERS-active layer was composed of silver nanoparticles as silver has good optical characteristics in a visible spectral range. Silver is the most suitable metal for creating SERS structures for the excitation by green laser radiation with a wavelength of 532 nm (Johnson and Christy, 1972). Recently, we have demonstrated that silver nanoparticle layer is unstable in biological fluids due to insufficient adhesion to the polymer surface (Kukushkin et al., 2022b). Hence, we applied a thin layer of chromium that improves the adhesive properties of the track membrane, allowing silver nanoparticles to adhere strongly enough to the surface. Moreover, it minimized the changes in the morphology of nanoparticles during the contact with biological fluids. Both types of the SERS-active membranes were used to filter influenza A virus samples labelled with the aptamers. It was shown that the increase in the silver nanoparticle layer stability, due to the addition of chromium, drastically decreased the intensity of SERS spectra (Kukushkin et al., 2022b). The presence of SERS spectra in the membranes with low adhesion to the polymer can be explained by the formation of aggregates of virus and several silver nanoparticles. These aggregates produce maximal SERS intensity due to a local increase of Q-factor. However, low adhesion to the membrane caused low reproducibility of the signal and spontaneous desorption of silver nanoparticles in the concentrated biological fluids. Thus, the in-crease of the adhesion is necessary for practical applications.

Here a new strategy has been proposed to provide SERS-based aptasensors using the SERS-substrate with good adhesion. The Raman-label was introduced into the aptamer that was attached to the surface of silver nanoparticles *via* a thiol group. The RHA0385 aptamer for influenza A virus was modified by thiol at the 5′-end and Cyanine-3 dye at the 3′-end. Adsorption of the labelled aptamer onto the SERS-substrate provided a SERS spectrum due to proximity of the label to the nanostructured surface. Initially, we used this aptamer to study the efficiency of adsorption of the aptamer onto the SERS-active substrate. Unexpectedly, we have explored that the aptamer could provide analyte-dependent changes of surface-enhanced Raman and fluorescence spectra. Although the detailed mechanism of the process is not clear; we suppose that influenza A viral particles orients the aptamers on the surface approaching the reporter to the surface (Figure 3). This orientation of the reporter increased both SERS and SEF spectra (Figure 7; Figure 8). Similar results were obtained for respiratory syncytial virus and aptamer to RSV labelled with TAMRA being in the agreement with suggested operating principle.

The size of pores is a crucial parameter for the membrane-based sensor performance. We have tested membranes with pore diameter of 30 and 800 nm with the same metal coating (Supplementary Figure S6). However, the robustness of the sensor was lost. Small pores did not filter out off-target biologicals, the pores were clogged, and the sensor provide the same SERS and SEF spectra for the samples of target and off-target viruses. The sensor with large pores did not detect influenza A virus at concentrations below 10^5^ VP/mL. Thus, the pore diameter is to be slightly bigger than the virus size that was 160 nm according to NTA data. Further optimization of the pore size and geometry is of high interest.

Specificity of the sensor was demonstrated by the absence of the increase of SERS and SEF in the presence of off-target influenza B virus. The limit of detection (LoD) was 10 VP/mL of influenza A virus for SERS spectrometry; SEF spectrometry provided 2.5-fold higher LoD, 25 VP/mL. The detailed comparison with LODs of re-ported SERS-based sensors for virus determination is provided in Table 1. The published works used a variety of influenza A strains and units for their quantification, so the comparison is rather ambiguous. For the direct comparison we used several reported correlations between different units of virus titre, including virus particles (VP), plaque forming unit (pfu) and median tissue culture infectious dose (TCID_50_) (Kramberger et al., 2012; Klimstra et al., 2020; McCormick and Mermel, 2021). The LoD of the proposed aptasensor of 10 VP/mL is much lower compared to antibody-based rapid tests (LoD = 1·10^6^–4·10^8^ VP/mL) and even PCR with reverse transcription (LoD = 3·10^2^–1.2·10^3^ VP/mL) (Herrmann et al., 2001; Chan et al., 2013; Klimstra et al., 2020). The LoD of the reported biosensor is the lowest among the published works (Table 1). Considering the minimal detectable quantity of viruses in the final probe, the proposed technique is close to PCR with reverse transcription, i.e. both tests are able to detect several viruses in the probe. The wide quantification range is an advantage PCR with reverse transcription; whereas, the aptasensor has limited facility for the virus quantification due to complex dependence of the signal on the viral titer; it is most useful for the virus identification purposes. An overall time of analysis of 15 min places the proposed technique in the raw of rapid test for point-of-care applications, whereas usage of the membrane allows concentrating from the large volumes. In addition, our approach requires fewer reaction steps due to the detection of a binary complex instead of ternary complexes or competitive assays. The single ‘wet’ procedure consists of the membrane filtration with simultaneous formation of the complex between the aptamer and the virus. This approach is promising for the practical implementation.

**TABLE 1:** Comparison of SERS or SEF-based sensors for the detection of viral particles with conventional diagnostic assays. N/A–not applicable, RT PCR–reverse transcription polymerase chain reaction, RT LAMP–reverse transcription loop-mediated isothermal amplification. * the recalculation was performed using the ratios from the refs. (Kramberger et al., 2012; Klimstra et al., 2020; McCormick and Mermel, 2021), **molecular weight of the influenza virus was approximated as 10^7^ Da.

Recog-nizing element	Virus type	Type of the SERS-substrate	Analytical performance			References
			Limit of detection	Quantifica-tion (yes/no)	Time of analysis	
None	Rheovirus, rinovirus, influenza A, parainfluenza	Gold nanoparticles onto carbon nanotube arrays	10^2^ EID_50_/mL (10^4^ VP/mL)*	No	15 min	Yeh et al. (2020)
	Respiratory syncytial virus	Silver nanorod array	100 pfu/mL (3·10^5^ VP/mL)*	Yes	1 h	Shanmukh et al. (2006)
	Circovirus, parvovirus, pseudorabies	Silver nanoparticles onto porous carbon films	1·10^7^ VP/mL	No	15 min	Luo et al. (2017)
Aptamer	Influenza A	Silver nanoparticles	2.2 · 10^−5^ HAU/mL (10^3^ VP/mL)*	No	15 min	Zhdanov et al., 2022b
		Gold nanopopcorn	97 pfu/mL (10^4^ VP/mL)*	Yes	20 min	Chen et al. (2020)
		Silver nanoislands onto silica oxide	5·10^–4^ HAU/mL (2·10^4^ VP/mL)*	No	12 min	Kukushkin et al. (2019)
		Silver nanoparticles	2·10^5^ VP/mL	Yes	15 min	Gribanyov et al. (2021)
		Silver nanoparticles onto the membrane	10 VP/mL	Yes	15 min	This work
		Gold nanopopcorn	1.06 HAU/mL (5·10^7^ VP/mL)*	Yes	15 min	Chen et al. (2022)
	SARS-CoV-2		0.95 pfu/mL (7·10^5^ VP/mL)*	Yess		
		Silver/Chromium/Gold lithographic nanocolumns	100 copies/mL (100 VP/mL)	Yes	15	Kukushkin et al., (2022a)
Antibody	Influenza A	Core-shell nanoparticles loaded with a dye	4·10^3^ TCID50/mL (4·10^5^ VP/mL)	Yes	3.5 h	Moon et al. (2016)
		Gold nanoparticles	30 ng/mL (1.8·10^8^ VP/mL)**	Yes	2 h	Lin et al. (2014)
	Human immunodeficiency virus	Gold nanostructured surface	35 pg/mL (2·10^5^ VP/mL)**	Yes	12 h	Lee et al. (2015)
RT PCR	Influenza A	N/A	3·10^2^–1.2·10^3^ VP/mL	Yes	2–3 h	Herrmann et al. (2001)
	SARS-CoV-2	N/A	10^2^–1.2·10^3^ VP/mL	Yes	2–3 h	Kim et al., 2022;
						Yang et al. (2021)
RT LAMP	Influenza A	N/A	10^4^ VP/mL	Yes	1 h	Luo et al. (2015)
	SARS-CoV-2	N/A	2·10^4^ VP/mL	Yes	1 h	Yan et al. (2020)
Anti-body-based test strip	Influenza A	N/A	1·10^6^ VP/mL	No	10–15 min	Chan et al. (2013)
			20 TCID_50_/mL			
	SARS-CoV-2	N/A	7.6·10^3^ TCID_50_/mL (5·10^8^ VP/mL)*	No	15 min	Author Anonymous (2021)

## 5 Conclusion

A novel type of a biosensor for virus identification has been proposed. The biosensor has several benefits over the standard types of sensors. For instance, it has a low cost (approximately 0.5 USD) and a high sensitivity (the LoD is 10–100 times lower than the LoD of RT PCR) requiring only a minimal number of steps. If we consider the market of rapid tests, then the main advantages of our sensor are a low price and excellent sensitivity. The main disadvantage in point-of-care application is requirement of Raman spectrometer. In our study, we used a portable autonomous Raman device. It could be made as cheap as $1,000 by using a simplified optical filtering and a detection scheme without registering a full spectrum a single reporter by rather focusing on a set of unique Raman lines.

## Data Availability

The original contributions presented in the study are included in the article/Supplementary Material, further inquiries can be directed to the corresponding author.
