# Supplementary material for: Aptamer-coated track-etched membranes with a nanostructured silver layer for single virus detection in biological fluids
Source: Front Bioeng Biotechnol. 2023 Jan 10;10:1076749. doi: 10.3389/fbioe.2022.1076749 (PMC9871243; doi:10.3389/fbioe.2022.1076749)
Supplement: Supplementary file 1 [file Table1.DOCX]

Supplementary Material


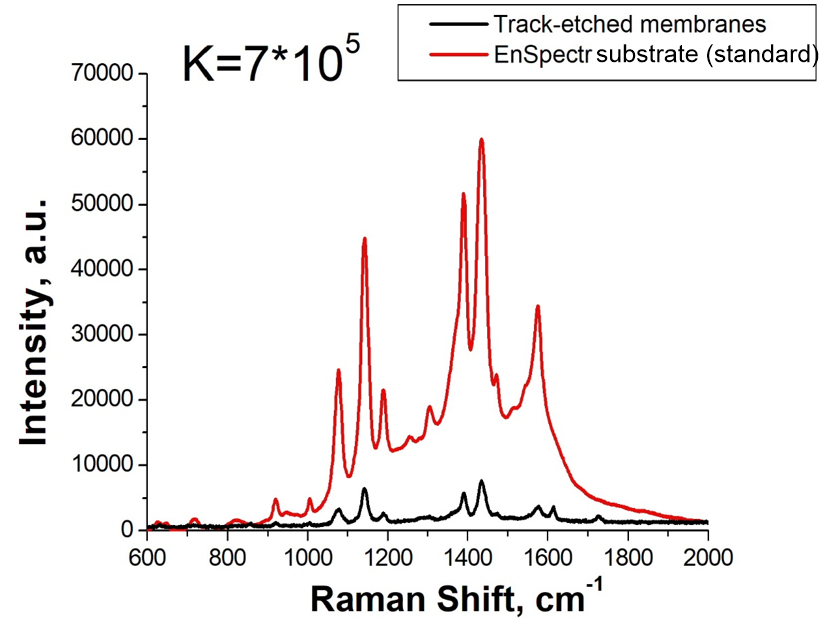


**Supplementary Figure S1.** Comparison of the Q-factor of the investigated membrane with a commercial substrate. K is a calculated Q-factor for the track-etched membrane.


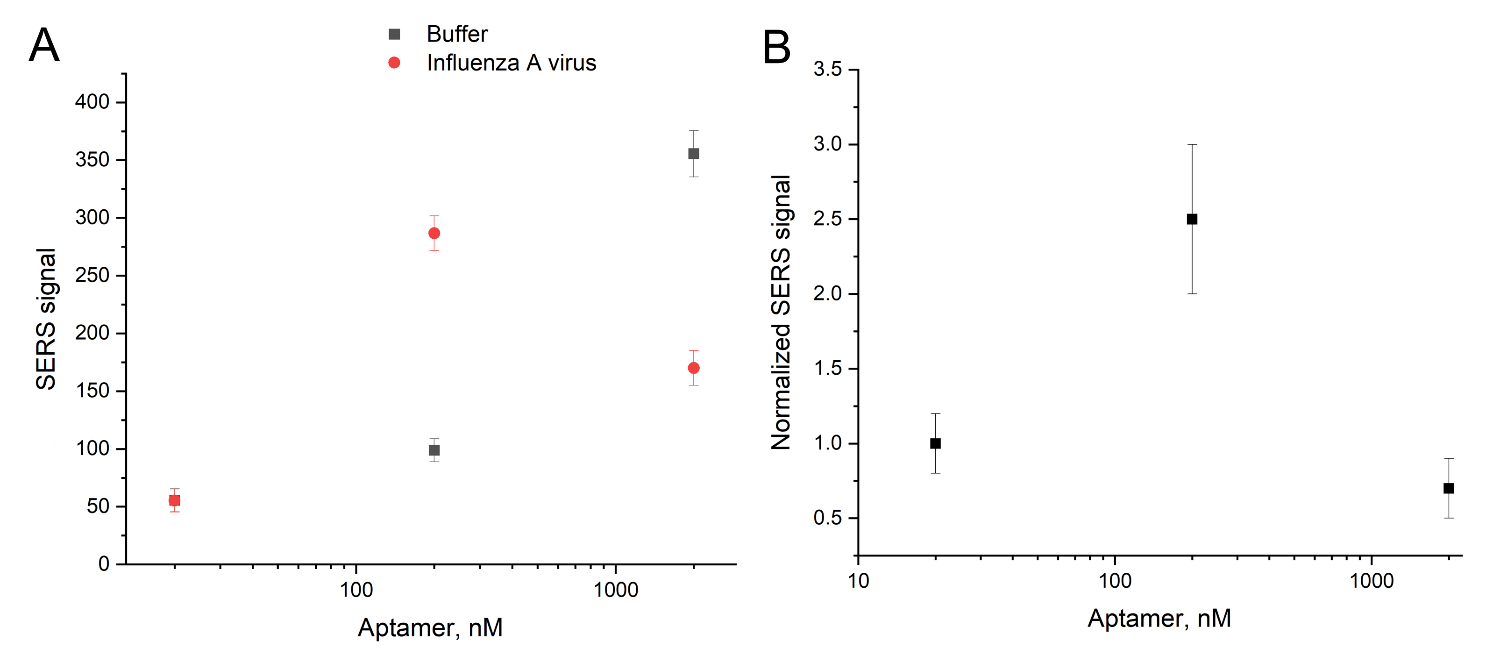


**Supplementary Figure S2.** The dependences of absolute (A) and normalized (B) SERS intensities on the concentration of the aptamer (20, 200 and 2000 nM) in the presence and absence of the influenza A virus in the concentration of 3·10^3^ VP/mL.


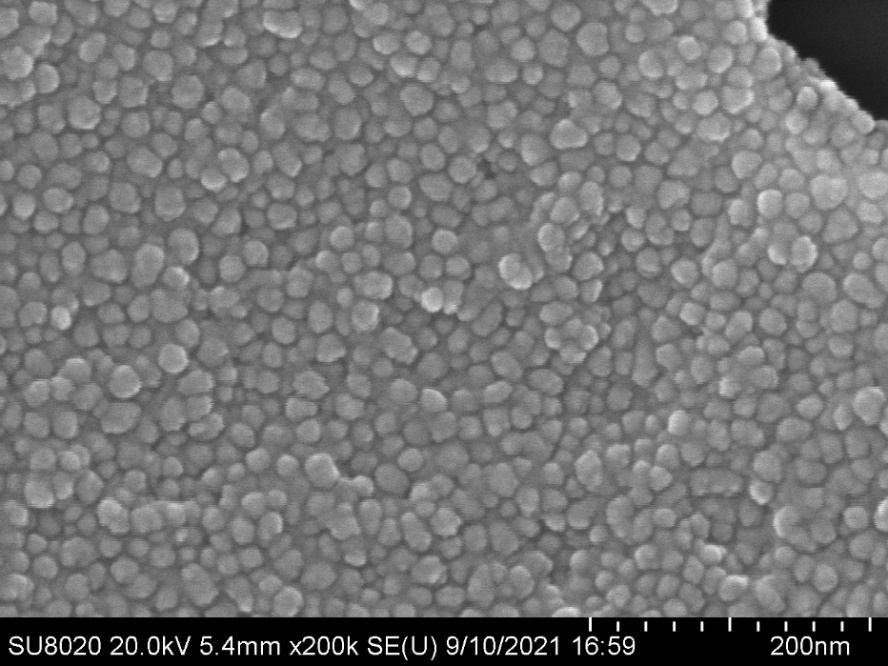


**Supplementary Figure S3.** Scanning electron microscopy of track-etched membranes functionalized with the aptamer before the filtration of PBS buffer. The surface topology is the same as for unfunctionalized membrane.


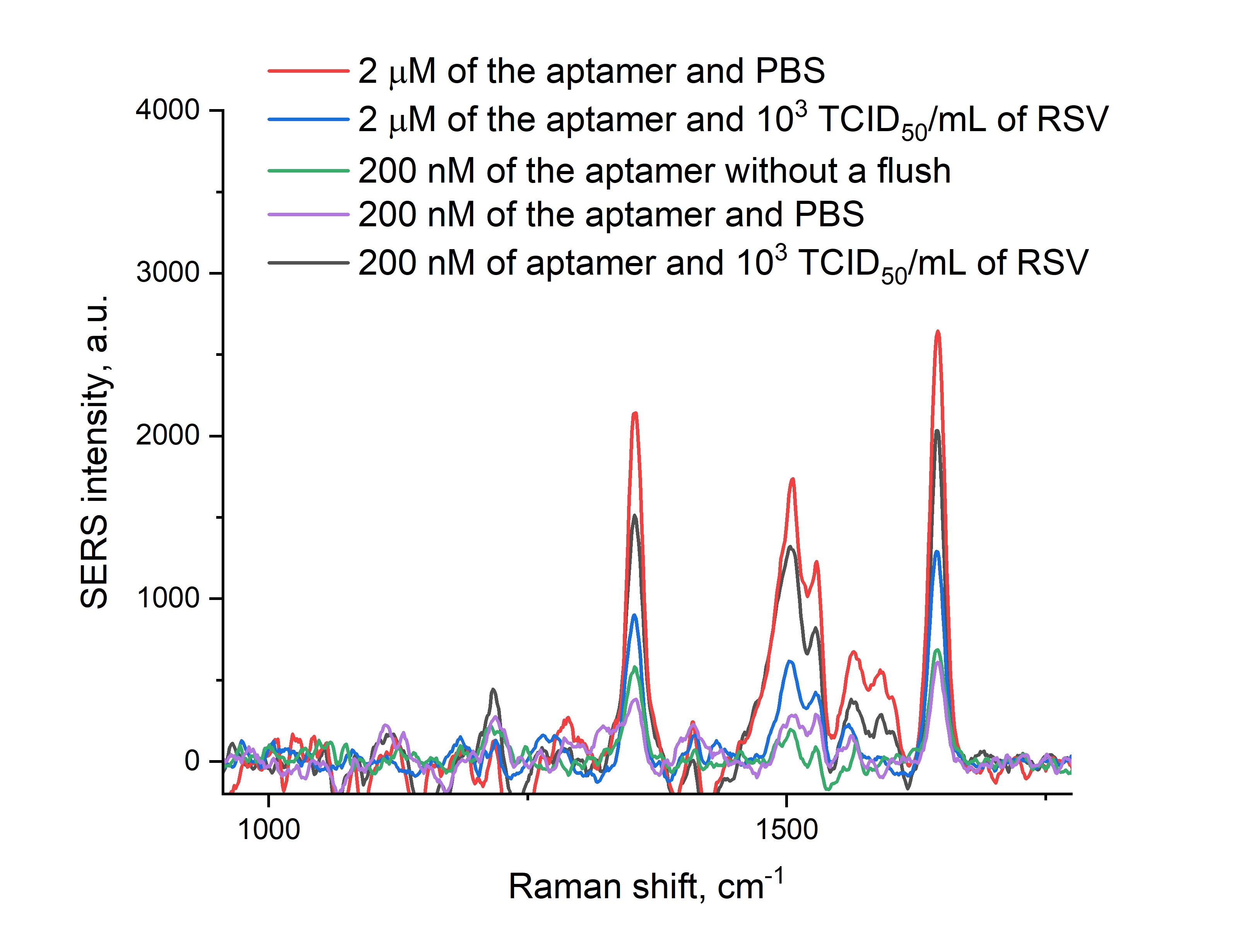


**Supplementary Figure S4.** SERS spectra of the membrane treated with 200 or 2000 nM of H8 aptamer to respiratory syncytial virus (RSV) in the presence and absence of RSV strain A2 in the concentration of 10^3^ TCID_50_/mL. The sequence of the aptamer was (SH)-5’-AGTGCGGTGA-GCCGTCGGACATACAAATAC-3’-TAMRA.


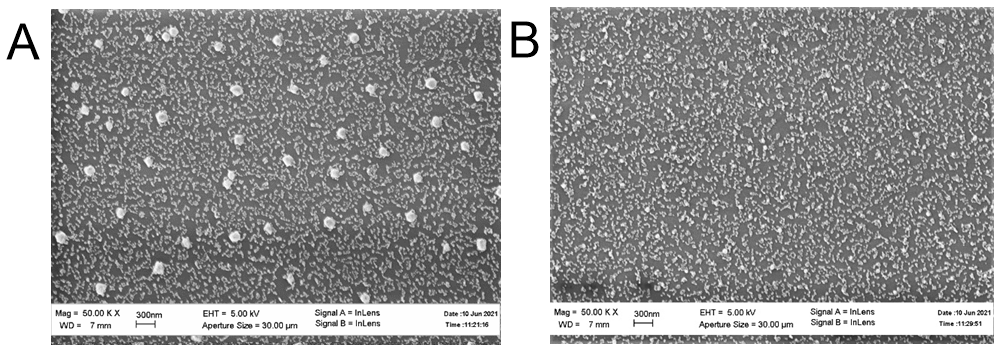


**Supplementary Figure S5.** Scanning electron microscopy of silver nanoislands onto silica oxide substrate after the functionalization with thiolated RHA0385 aptamer and the interaction with 2·10^6^ VP/mL of influenza A virus (A) or 2·10^6^ VP/mL of Newcastle disease virus (B). The technique of the aptasensor was the same as in the work (Kukushkin et al., 2019). Scanning electron microscopy was performed without an additional deposition of metals, so the bright objects with 100 nm correspond to the aggregates on silver nanoparticles that presumably surround influenza A viruses. The size of the aggregates corresponds to the size of influenza virions (nearly 130 nm).


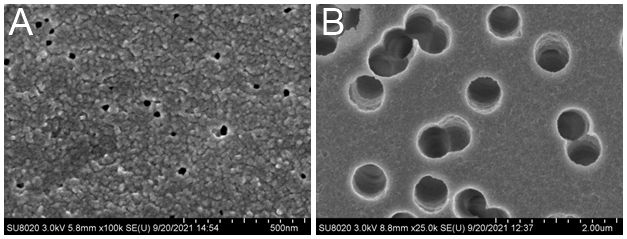


**Supplementary Figure S6.** Scanning electron microscopy of track-etched membranes covered with silver-chromium layer. The pore diameter was 30 nm (A) or 800 nm (B).

**References**

Kukushkin, V.I., Ivanov, N.M., Novoseltseva, A.A., Gambaryan, A.S., Yaminsky, I.V., Kopylov, A.M., Zavyalova, E.G. (2019). Highly sensitive detection of influenza virus with SERS aptasensor. *PLoS One* 14, e0216247.
